# Effect of Zn and Mg Content on Crashworthiness of Al-Zn-Mg Alloy Thin-Walled Square Extrusions

**DOI:** 10.3390/ma13214791

**Published:** 2020-10-27

**Authors:** Hui Guo, Cheng Wang, Jin Zhang, Yunlai Deng

**Affiliations:** 1 Light Alloy Research Institute, Central South University, Changsha 410083, China; guohuijames@163.com (H.G.); peterchenglari@csu.edu.cn (C.W.); 2 State Key Laboratory of High Performance and Complex Manufacturing, Central South University, Changsha 410083, China; luckdeng@csu.edu.cn; 3 School of Materials Science and Engineering, Central South University, Changsha 410083, China

**Keywords:** Al-Zn-Mg alloy, extrusion, crashworthiness, mechanical property, axial compression

## Abstract

The effects of Zn and Mg content in thin-walled square extrusions of Al-Zn-Mg alloys on its crashworthiness were investigated, and the correlation between the crushing properties, mechanical properties, and microstructures of the profiles were investigated. The results showed that the strength and the compression properties were gradually increased with a decrease in the Zn/Mg ratios (from 12.48 to 4.57). When the Zn/Mg ratio is lower (less than 6.29), an increase in the Mg content simultaneously improves the alloy strength and the compression properties. An increase in Zn content (from 5.07 to 6.77) can improve the strength of the alloy however, it does not affect the compression properties. However, the higher Zn contents (6.77%) would lead to cracking in advance during the compressing, which reduces the compression energy absorption capacities of the product. Therefore, in order to obtain higher strength and excellent compression properties, the Zn/Mg ratio should be reduced. For the upper limit, the Zn content should not be too high (less than 6.77), as this may lead to early cracking and failure. For the lower limit, the Mg content should be higher (more than 0.91) to make sure that the alloy has excellent compression properties and higher strength.

## 1. Introduction

Owing to the high energy absorption and weight efficiencies of thin-walled aluminum alloy extrusions with cavity sections, they are often used as collision-energy absorption components in vehicles. The extrusion profile absorbs the collision energy by axial crushing deformation, and thus plays a buffering role by effectively protecting the passengers and the body of the vehicle [1,2,3].

Several experimental and numerical studies have been conducted to optimize the energy absorbing capacity of aluminum alloy extrusion profiles under axial crushing. Most of these studies have focused on obtaining an optimum structural design. Cheng et al. [4] studied the crush behavior of 6061-T6 aluminum alloy square tubes. They showed that the total energy absorbed by the aluminum alloy square tubes could be increased to be within a range of 26.6–74.6% by introducing optimization through-hole discontinuities. Sun et al. [5] found that the increasing number of corners on the cross-section of multicorner tubes improved the energy absorption performance of 6060 aluminum alloy thin-walled square tubes. They obtained a maximum crushing force efficiency of 11.6%, which was higher than that of the square tubes made with the same material. Kohar et al. [6] developed optimized sectional sizes of extrusion profiles using a multicellular 6063-T6 aluminum alloy with finite elements and neural network metamodeling. The new profile had a higher mean crush force that showed a 26.7% increment in energy absorption than that in the initial size. Acar et al. [7] evaluated 30 different multicell design concepts to optimize the crash performances of thin-walled aluminum tubes. Their results showed that the optimal design was nearly twice as high as the worst design.

Researchers are now focusing on the selection of appropriate materials to develop alloys and on process optimization. Granum et al. [8] investigated the mechanical properties, microstructural parameters, and the axial crushing performances of rectangular hollow section profiles made from AA6063, AA6061, and AA6110 aluminum alloy in T6, T7, and O tempers. A nanostructure-based finite element simulation model was developed and showed excellent prediction of the experimentally obtained force-deformation curves as well as the energy absorption capacity. Hoang et al. [9] studied the effect of different types of cooling after a solid solution on the axial crushing performance of AA6060 aluminum alloy square hollow section profiles. They observed that the effect of the cooling rate on the absorption of the crushing energy of the under-aging and over-aging profiles was insignificant, but it has a great influence on the peak-aging profiles. Moreover, the profiles with lower cooling rates absorbed less energy.

**Figure 1:**
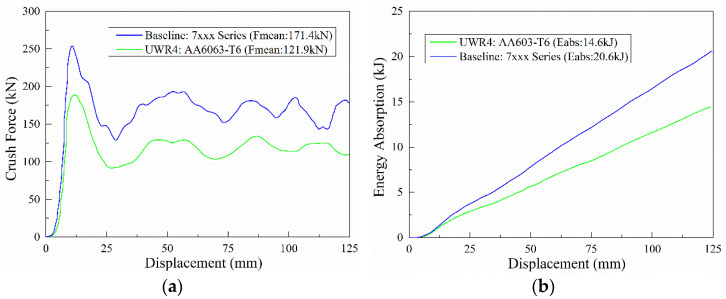
Experimental (**a**) fore-displacement and (**b**) energy response for baseline profile-7xxx series and UWR4 profile-6063-T6.

Commercially available Al-Zn-Mg alloys with little or no copper content have very high specific strengths and show good weldability [10,11,12,13,14]. An application of these alloys in the manufacture of lightweight vehicles has garnered increased attention in recent years and they have been applied in high-speed trains and civil automobiles. There is a need to improve the safety components of the vehicle to enhance their collision-energy absorption capacities. Presently, Al-Zn-Mg alloys (7xxx series) with higher specific strengths are becoming more popular than Al-Mg-Si alloys (6xxx series), which are the most widely used. Figure 1a shows a comparison of the force–displacement curves of a common 7xxx alloy profile with that of an optimized 6063-T6 alloy profile. Clearly, the energy absorption level of the 7xxx alloy profile is higher, probably because the extrudability of the Al-Zn-Mg alloy was inferior to that of Al-Mg-Si alloy. The application of Al-Zn-Mg alloys for mass-production practices over the past has been limited. Therefore, past studies on the crashworthiness of aluminum alloy extrusion have mainly focused on Al-Mg-Si alloys with little or no emphasis on Al-Zn-Mg alloys. However, the number of studies to understand the mechanism of the extrusion processes and microstructures of Al-Zn-Mg alloys for application in vehicles has gradually increased, and this has significantly improved the properties of the extrusion profiles [15,16,17,18,19,20]. Chen et al. [21] found that the tensile strength, stress corrosion cracking, and electrochemical corrosion resistance can be significantly increased with a decrease in the Zn/Mg ratio in the Al-Zn-Mg alloys with Zn + Mg ≈ 7 wt.%. In addition, Chen et al. [22] showed that the addition of 0.5 wt.% Mg can enhance the strength of Al-Zn-Mg alloys with high Zn concentration after rolling and heat treatments owing to the presence of high-density nano-sized Zn and η′ phases. As suggested by Chen et al., the material properties of Al-Zn-Mg alloys can be further improved by optimizing its composition. Further research is needed to understand the effects of adjusting the principal alloy compositions on other properties of the extrudates.

To further improve the crashworthiness of Al-Zn-Mg, the main alloy compositions may be modified within a given specification. This study is aimed at optimizing the composition of the main compositions in Al-Zn-Mg alloy thin-walled square extrusions to improve its crashworthiness. Several microstructural analyses were performed and the basic mechanical properties and crushing performance of Al-Zn-Mg alloy extrusions with different ratios of the main compositions in the alloy were investigated.

## 2. Experimental

**Figure 2:**
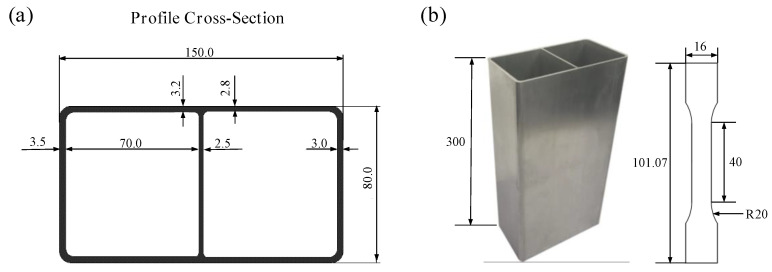
(**a**) Profile cross-section geometry diagram of the extrusions and (**b**) geometry of extruded workpiece and tensile specimens.

Five different Al-Zn-Mg alloys are investigated in this study, the compositions shown in Table 1. The raw materials were received as homogenized ingots with a 230 mm diameter. The ingots were heated to 510 °C and extruded into the rectangular hollow section profiles with double chambers with a constant ram speed of 5 ± 0.2 m/min, as shown in Figure 2. The extruded profiles were water-mist quenched on line from the outlet of the die, while the outlet temperatures were held constant at 565 °C ± 2 °C and then lowered close to ambient temperature through the air-cooler. Subsequently, the profiles were immediately moved in the furnace and subjected to two stages of artificial aging 5 h at 105 °C and 18 h at 155 °C. Afterwards, the profiles were cut into lengths of 300 mm for the quasi-static axial crush testing. Approximately, the first half meter of the extruded profile for each alloy was discarded due to possible contaminants.

**Figure 3:**
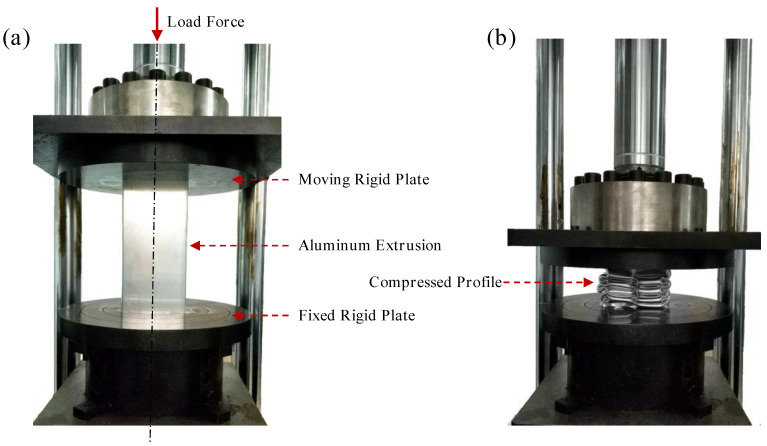
(**a**) Compression test bench structure and (**b**) compressed profile of the workpiece.

Uniaxial tensile tests were conducted for all 5 alloys employing a CSS–44100 testing machine (Jinan Ruima machinery Equipment Co. LTD, Jinan, Shangdong, China) at a tensile speed of 2 mm/min. The tensile specimens were taken from the artificial aged profiles along the extrusion direction, and the geometry of the specimens is given in Figure 2b. During the tests, an extensometer was used to monitor the deformation of the specimens. Three parallel tests were carried out for each alloy. For the stability of the extrusion process itself, the spread among three parallel tests was insignificant. And the test experiencing median force level within each alloy sample was chosen as the representative stress-strain curve. To study the energy absorption capability of the five different extrusion profiles, quasi-static axial crushing tests were performed at ambient temperature, by using WAW-100FC hydraulic universal testing machine (Shandong Aubert Test Equipment Co. LTD, Jinan, Shangdong, China), as shown in Figure 3. Specimens were placed on the loading center below a fixed horizontal plate and no constraints were imposed. The upper plate moved vertically downward at a speed of 100 mm/min, and the final displacement was 200 mm during the test. The axial stress and displacement history were recorded. After the crush experiments, the microstructure of the characteristic parts were observed by optical micrographs (OM) (Shanghai Puhe Optoelectronics Technology Co., LTD, Shanghai, China) and spliced to form a more complete cross-sectional micrographic picture. The recrystallized structure of characteristic parts were observed and analyzed by electron back scattered diffraction (EBSD) (ThermoFisher Scientific, Waltham, MA, USA).

## 3. Results

### 3.1. Mechanical Properties of Al-Zn-Mg Alloy Extrusions with Different Main Compositions

**Figure 4:**
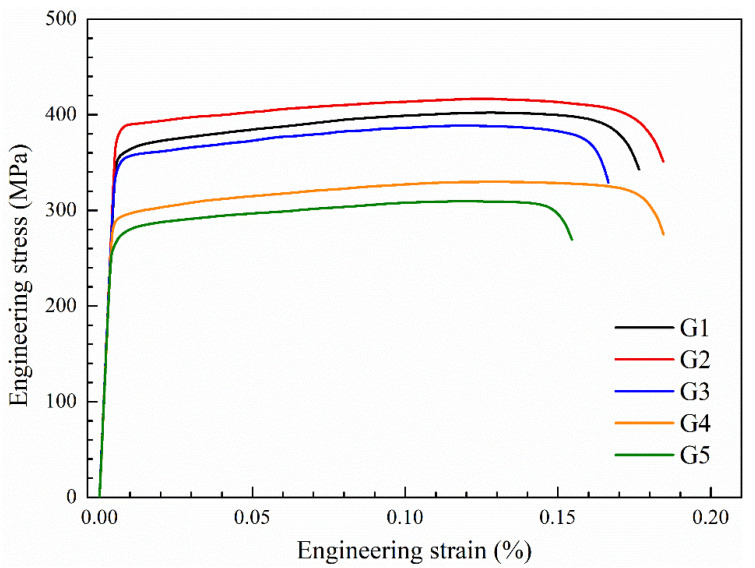
Engineering stress-strain curves of Al-Zn-Mg alloy extruded profile with different main compositions for T73 temper.

The typical engineering stress-strain curves of the T73 specimens of the five Al-Zn-Mg alloys extrusions with the different main compositions are shown in Figure 4. The mechanical properties of the profile samples with different main compositions made by the same process were significantly different, in which the yield strength ranged from 270 MPa to 400 MPa and the tensile strength ranged from 310 MPa to 431 MPa. For the five samples studied in this study from G1 to G5, as the Zn/Mg ratio gradually increased, the strength of the samples showed a gradual decreasing trend, and the elongation remained at a relatively high level (≥15%). Among them, the G2 sample does not conform to the general law, and its strength and elongation are obviously higher than the G1 sample. Comparing the G1 and G2 alloys, the Mg content is similar and the Zn content is different. The Zn content of the G2 alloy is 1.7 wt.% higher than that of the G1 alloy. It can be seen that the increase of Zn content can simultaneously improve the strength and elongation of Al-Zn-Mg alloy at room temperature. Comparing the G3 and G4 alloys, the Zn content is similar and the Mg content is different. The Mg content of the G3 alloy is 0.26 wt.% higher than that of the G4 alloy, which increases the yield strength of the alloy by 59 MPa and the tensile strength by 58 MPa, but reduces the elongation by 1.8%. Although there was little change in Mg content and little difference in the total amount of Zn + Mg, there was a large difference in the ratio of Zn/Mg (G4 increased by about 40% compared with G3). Comparing G2 and G3 alloys, Zn/Mg ratios was similar but the total amount of Zn + Mg increased, and the content of Zn and Mg increased respectively. According to the effect laws of Zn and Mg mentioned above, the strength of the G2 alloy should be enhanced superimposed, which is consistent with the results of the stress-strain curve shown in Figure 4. This also confirms the effect law of Zn and Mg content on the mechanical properties of Al-Zn-Mg alloy obtained above.

### 3.2. Crushing Properties of Al-Zn-Mg Alloy Extrusions with Different Main Compositions

**Figure 5:**
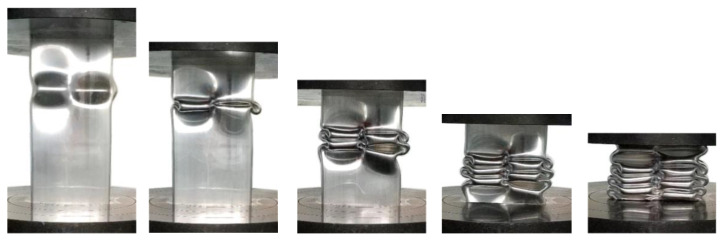
Consecutive stages of collapse of the G3 extruded profile sample under compression test.

**Figure 6:**
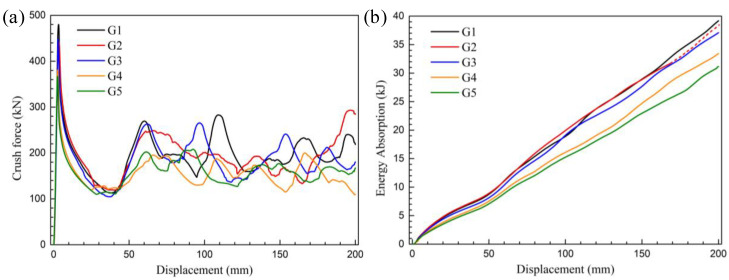
During the axial compressing test of Al-Zn-Mg alloy extrusions with different main compositions: (**a**) The force-displacement curves and (**b**) energy absorption curves.

The five samples studied in this study all have high elongation (≥15%). They have no obvious rupture during axial crushing, and roughly maintained the deformation integrity during the compressive process, as shown in Figure 5. The first folding deformation of the Al-Zn-Mg profile occurs at an about 70% height near the top of the sample, and the direction of folds alternates between the two chambers and an asymmetrical folding pattern is formed. Although there were slight differences in subsequent folding process, global good repeatability was shown. By observation of the crushed samples, it can be found that the other samples good folding without fracture except for a ductile crack formed at the middle corner of the G2 sample. Figure 6a depicts measured force-displacement curves during the axial crushing test of Al-Zn-Mg alloy profile samples with different main compositions. The number of peaks of the force–displacement curves exactly coincides with the number of folds. One of the characteristics of this curve is that the displacement point and duration of the first peak of all samples are close to each other, except for the difference in the peak load, and the subsequent stress peaks gradually become disordered. Another characteristic is that the five different alloys designed in this study have a very high initial peak load, ranging from the lowest value of 372 kN (G5 sample) to the highest value of 480 kN (G1 sample), which reflects the limit value of the bearing capacity of thin-wall extrusion structures. During the global crush testing, the first stress peak corresponds to the plastic buckling stage of the first fold. Since the first peak load and displacement account for the largest proportion in the entire crush test process, it also reflects the crushing energy absorption capacity of the sample to some extent. Figure 6b shows the relationship between energy absorption and displacement during the axial crushing test of the Al-Zn-Mg alloy extrusions samples with different main compositions. The energy absorption *E*_ab_ can be obtained by integrating the stress (F)—displacement (S)—curve, as follows:

(1) $$E_{\mathrm{ab}}=\int_{0}^{S}F\left(S\right)\mathrm{dS}$$

It can be seen from the comparison of the energy absorption curves that the final total energy absorption from samples G1 to G5 decreases sequentially with the Zn/Mg ratios increases. In the energy absorption curves, G1–G3 and G4–G5 showed a large interval, and there is a similar phenomenon on the tensile curve. The G3–G5 alloy is located at the lower level of energy absorption curve, and shows the law of monotonously decreasing from the beginning. Therefore, among the three alloys, the alloy composition of G3 has an absolute advantage in energy absorption by compression. In G1–G3 alloys with a high energy absorption when the crosshead goes down to the end point of 200 mm displacement, the G2 alloy needs to be emphatically discussed due to the non-monotonic changing characteristics of the G1 and G2 alloy and the rupturing phenomenon of the G2 alloy in the near end of compression.

## 4. Discussion

### 4.1. Design Boundary Conditions of the Main Compositions to Improve the Crushing Resistance of Al-Zn-Mg Alloy Extrusions

During the crushing process, the G2 alloy was ruptured when the compression displacement reached 152.7 mm, corresponding to which the load caused by the tearing material accumulation increasing rapidly and exceeding the previous peak value, as shown in the red curve in Figure 6a. In practice, when this happens to the energy-absorbing parts, adjacent auto parts will be damaged, thus losing their energy-absorbing and protective effects. Therefore, this energy absorption due to material accumulation is invalid and cannot be counted in the total energy absorption of the G2 alloy. In Figure 6b, the energy absorption curve of the G2 alloy in the post–collapse is marked as a dashed line and only the solid line is included in the final crushing performance results listed in Table 2.

**Figure 7:**
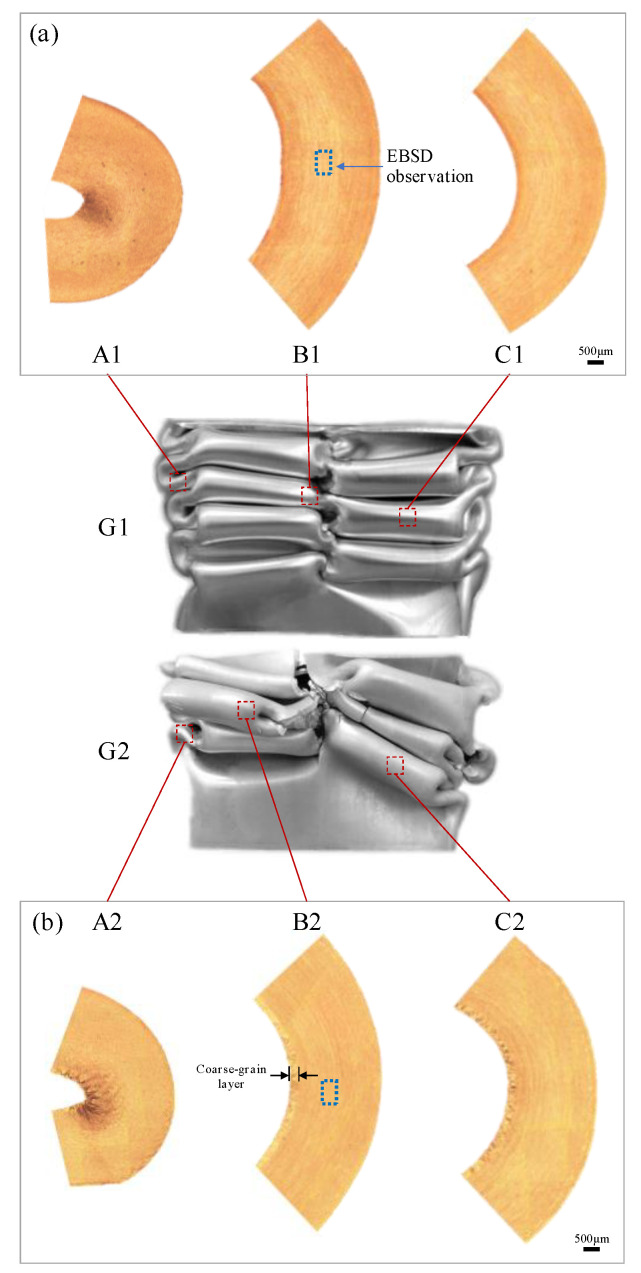
Metallographic micrographs of the compressed specimen fold area: (**a**) G1 sample; (**b**) G2 sample.

**Figure 8:**
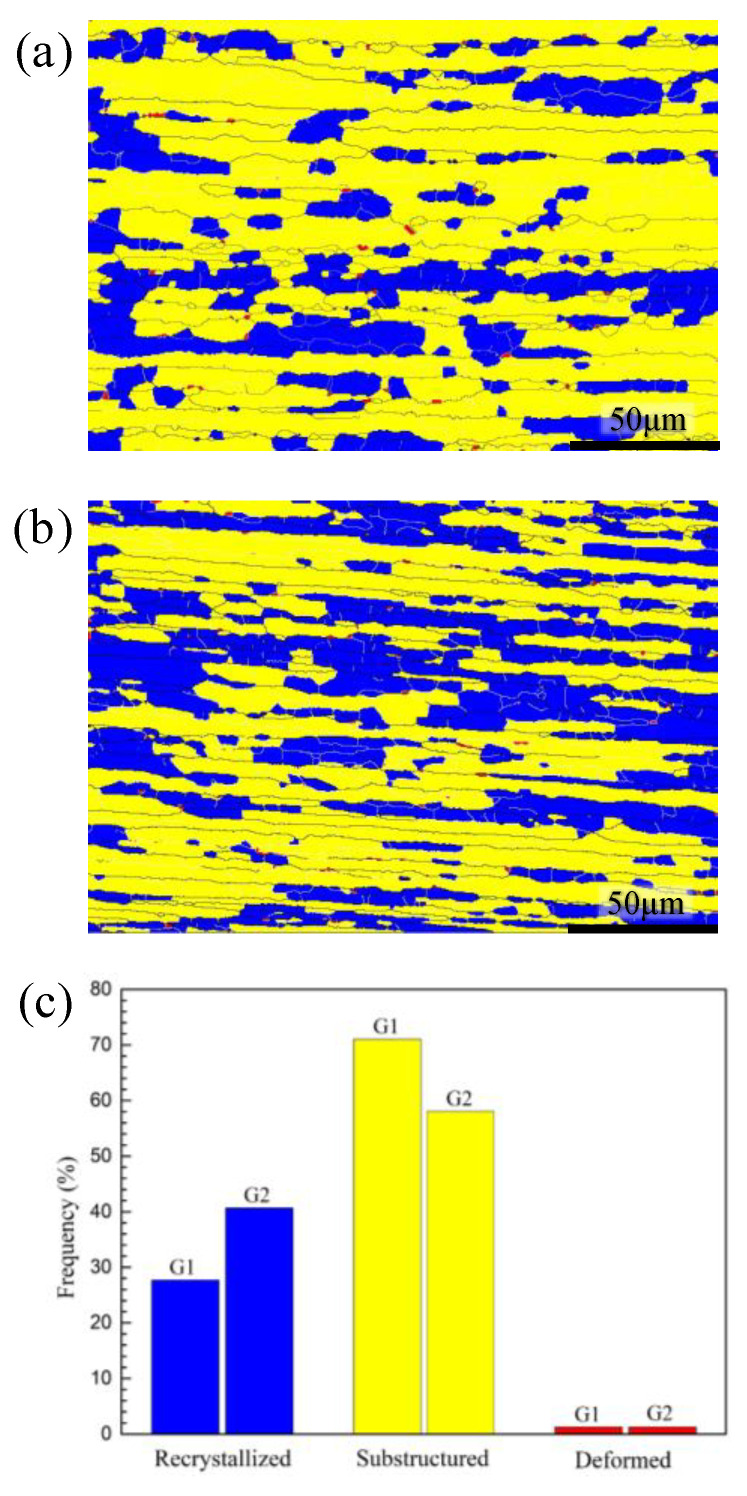
The distribution of recrystallized grains, sub-grains, deformed structure in the electron back scattered diffraction (EBSD) maps of the central layer for G1 and G2 samples: (**a**) G2 samples; (**b**) G2 samples; and (**c**) percentage statistics.

**Figure 9:**
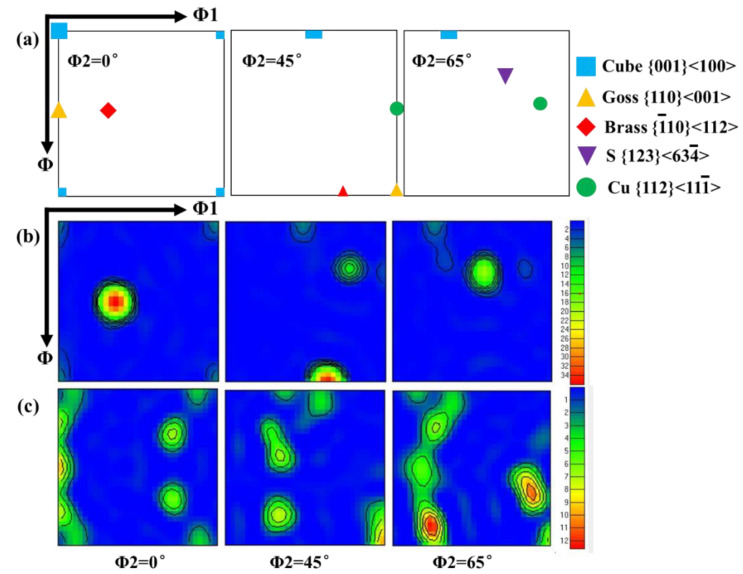
Φ2 = 0, 45, and 65° sections of orientation distribution functions (ODF) maps of Al-Zn-Mg alloy extrusions: (**a**) Marked locations of texture components; (**b**) G1 samples; and (**c**) G2 samples.

**Figure 10:**
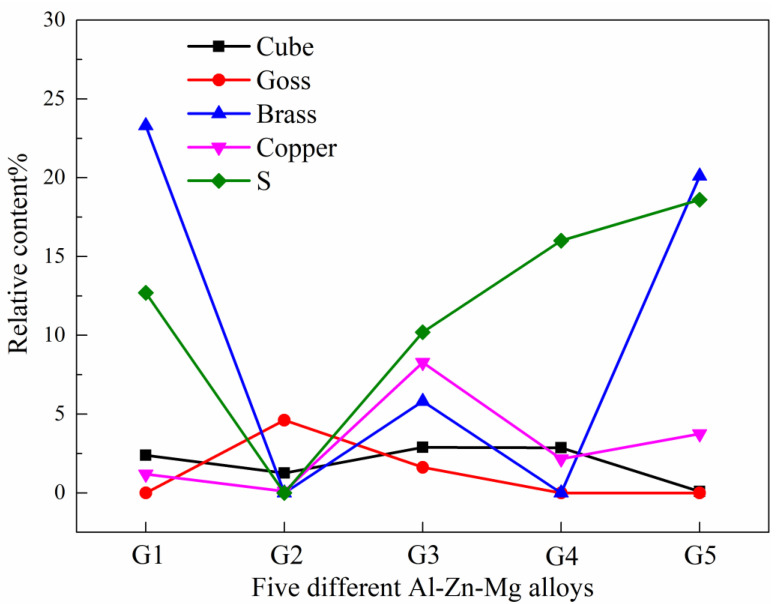
The main textures relative content in the five different layers.

By comparing the cross-section micrographs microstructure of the characteristic area of G1 and G2 samples after crushing tests (as shown in Figure 7), it can be found that the surface layer in the sample cavity of G2 alloy extrusions has an obvious coarse grain ring (thickness about 0.3 mm). This is because the inner wall of the hollow cavity has a more severe material flow during the crushing process, the friction is greater between the material surface and mold. This difference between G1 and G2 alloys under the same extrusion process indicates that when the Zn/Mg ratios is low and the Mg content is similar, G2 alloys with a higher Zn content have a higher tendency of recrystallization during hot deformation. This tendency can also be reflected by comparing the recrystallization fraction in the EBSD analysis results of the central layer of G1 and G2 alloy samples shown in Figure 8 (the samples are taken from the box marked positions of B1 in Figure 7a and B2 in Figure 7b, respectively). The recrystallization fraction of the central layer of G2 alloy extrusions is relatively higher. Figure 9 shows the orientation distribution functions (ODF) of G1 and G2 alloy samples (φ2 = 0, 45, and 65°). Figure 9a shows the position labeling of major texture components in ODF. Comparing the G1 and G2 alloy, the texture intensity of the G2 sample was significantly weakened due to the relatively higher recrystallization of G2 alloy as shown in Figure 9b,c. Among them, goss texture intensity was increased, whereas intensities of brass texture and S texture were significantly weakened, while cube texture and copper texture have little difference. In order to distinguish and quantify the texture components characteristics of different main component alloys more clearly, the texture components of five different alloys was statistical analyzed as shown in Figure 10. The texture in G1 sample was dominated by a strong brass texture and S texture, while the texture in G2 sample was only the weak goss texture. The texture in G3 sample was composed of strong brass, copper, and S texture components. The texture in G4 sample was dominated by the strong S texture, while the texture in the G5 sample was dominated by a strong brass texture and S texture. These results illustrated that different main compositions can affect the texture composition and intensity of Al-Zn-Mg extrusions. The textures in the G2 sample almost only exists recrystallization textures, while deformation texture was dominant in G1, G3, G4, and G5 samples. Obviously, the above- mentioned coarse grain ring is prone to generate microcracks when it is compressed and folded. It can be seen that a large number of microcracks are gathered in the inner wall coarse grain ring of the G2 alloy with a large folding deformation. It can be seen in the A2 region with the largest folding that this aggregated microcracks have propagated into the internal grain microstructure. According to the simulation research results of Hartmann Matthias et al. [23] on this double–cell rectangular thin-wall extrusions, the maximum tensile stress occurs in the inner wall of the corner region of the middle T-shape during the folding process. It was safe assumption that the coarse grain ring in this area must preferentially aggregate the cracks, resulting in rupture from the middle of the G2 alloy extrusions.

**Figure 11:**
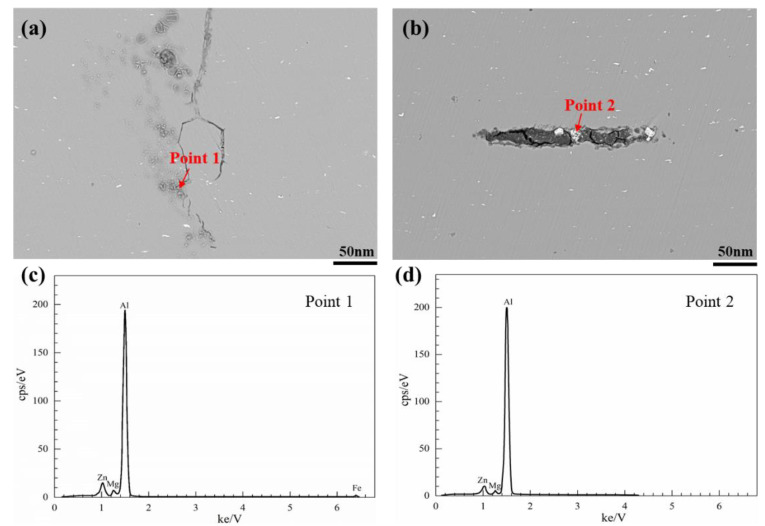
SEM photographs of cracking areas of compressed G2 specimens (**a**) and (**b**), and EDS analysis results (**c**) and (**d**).

According to the test results of tensile mechanical properties, the crush energy absorption capabilities and strength properties of the five different alloys in this study are roughly consistent. It is a special case that the G2 alloy fails due to early cracking. The five alloys with relatively high elongation will not undergo brittle fracture. In this study, the forming and testing conditions as well as the sample structure are as consistent as possible, which can eliminate the factors such as the structure and process defects of the profiles. Since the strength and elongation of the G2 alloy are the highest levels of the five samples, it can be inferred that the cracking is most likely due to the local weakening of the microstructure at the stress concentration area. SEM observation and second-phase composition analysis were conducted near the cracking source of the alloy, as shown in Figure 11. It was found that there were more residual eutectic phases of main alloy compositions in G2 alloy. The eutectic phase is easy to accumulate at the grain boundary when the main alloy compositions is increased, and it is easy to produce dislocation plie-up when crushing results in severe cold deformation, and then develop into crack source, lead to cracking. Although the residual eutectic phase can be improved by heat treatment such as enhanced homogenization and enhanced solid solution, this cracking tendency of the designed alloy still brings a safety hazard. We have also confirmed in repeated experiments that the collapse of G2 alloy samples is not accidental. Therefore, from the perspective of the energy absorption capabilities obtained and risk, the G2 alloy is not recommended as the main compositions design of crushing materials, despite its excellent tensile strength and elongation. To some extent, this can also be used as the upper limit of the main compositions design of the energy absorption Al-Zn-Mg alloy towards high strength direction. In conclusion, Al-Zn-Mg alloys with a higher strength will not be suitable crushing energy-absorbing structural materials.

### 4.2. Optimize the Main Compositions of Crushing and Energy Absorption of Al-Zn-Mg Alloy Extrusions

Apart from the G2 sample, the Zn/Mg ratios increased gradually from G1 to G5, and the total energy absorption decreased gradually, and the same size relationship was maintained throughout the crushing process. Moreover, the nominal yield strength and tensile strength of the samples were subsequently reduced, consistent with the research results of Sonyi Chen et al. [21], who have demonstrated that this is caused by changes in the volume fraction of the precipitated phase in the matrix, and that the Zn/Mg alloys with lower values also have better corrosion resistance. It can be seen that controlling the Zn/Mg ratios is a good compositions design direction for obtaining excellent properties of Al-Zn-Mg alloy extrusions.

Certainly, the results of this study are more than the above conclusions. A lot of useful information can be obtained by pairing between samples. For example, by comparing G1 and G2 samples, it can be found that the Mg content of the two alloys is similar but the Zn content is different. The increase of Zn content can obviously improve the strength of the alloy, but it has no effect on energy absorption. In addition, more non-equilibrium eutectic phase will be produced, leading to cracking and reducing energy absorption capacity. By comparing the samples of G3 and G4, it can be found that the Zn content of the two alloys is similar but the Mg content is different. The increase of Mg content can not only improve the strength of the alloys, but also significantly increase energy absorption. The research by Yunlai Deng et al. [24] shows that the supersaturated solid solubility of Al-Zn-Mg alloy matrix increase with an increase of Mg content and make the ranges of precipitation temperature wider, so the quenching sensitivity was higher. This is disadvantageous to the continuous production of extrusion on line. From the above analysis, it can be seen that the G1 alloy designed in this study is a recommended main composition range.

## 5. Conclusions

In this study, the effects of Zn and Mg content on the energy absorption capacities of thin-walled square extrusions of Al-Zn-Mg alloy were investigated through axial static crushing tests and tensile tests at room temperature. OM, SEM, and EBSD microstructure analyses were conducted. Based on the results obtained, the following conclusions are drawn:

(1) The strength and crushing energy absorption capacity of the samples from G1 to G5 changes were similar and they generally showed an increasing trend with a decrease in the Zn/Mg ratios. The yield strength ranged from 270 MPa to 400 MPa, and the tensile strength ranged from 310 MPa to 431 MPa. The first peak value of the crushing stress from the lowest value of 367 kN to the highest value of 480 kN;
(2) When the Zn/Mg ratio is relatively low, an increase of Zn content can improve the strength of the alloy however, this does not affect the crushing energy absorption capacity, while an increase in the Mg content can simultaneously improve the strength of the alloy as well as the crushing energy absorption capacity. The G1 alloy has the highest total crush energy absorption capacity and the G2 alloy has the highest strength. However, the G2 alloy, with a relatively high Zn content, has a higher recrystallization tendency during hot deformation. Consequently, a coarse grain ring is formed and many non-equilibrium eutectic phase is produced, and this leads to cracking in advance during crushing as well as a reduction in the energy absorption capacity;
(3) Controlling the Zn/Mg ratio (within the range 4.57 to 6.29) is a good composition design direction for obtaining an excellent comprehensive performance of Al-Zn-Mg alloy extrusions. As a crushing resistant structural material, from the perspective of risk and obtained energy absorption capacities, the Zn/Mg ratio should be reduced in the high strength direction of the design of the main compositions however, the upper limit during selection is that the Zn content should not be too high as this may lead to early cracking and failure (e.g., in case of the G2 alloy). The lower limit during selection is a higher Mg content to ensure excellent crushing properties and higher strength (e.g., in case of the G1 alloy).

## Figures and Tables

**Table 1:** Chemical composition of Al-Zn-Mg alloys (mass fraction/%).

Sample No.	Si	Fe	Cu	Mg	Zn	Zr	Al	Zn/Mg	Zn + Mg
G1	0.05	0.08	0.15	1.11	5.07	0.16	Bal.	4.57	6.18
G2	0.04	0.09	0.16	1.10	6.77	0.16	Bal.	6.15	7.87
G3	0.05	0.08	0.16	0.91	5.73	0.16	Bal.	6.29	6.64
G4	0.04	0.08	0.15	0.65	5.72	0.15	Bal.	8.80	6.37
G5	0.04	0.09	0.16	0.52	6.49	0.15	Bal.	12.48	7.01

**Table 2:** Crushing properties test results for Al-Zn-Mg alloys thin-walled square extrusions with a different main composition.

Sample	Energy Absorption	Peak Crush Force	Mean Crush Force
	(kJ)	(kN)	(kN)
G1	39.18	480.1	204.2
G2	31.18	440.9	195.9
G3	37.09	448.4	185.5
G4	33.43	380.7	167.2
G5	31.16	367.2	155.8
